# Gender Matters: Identity, Risk Perception and Preventive Interventions for Alcohol Consumption among Adolescents Using a Qualitative Approach

**DOI:** 10.3390/ijerph192416435

**Published:** 2022-12-07

**Authors:** Lucía Martínez-Manrique, Maitane Berasaluce, Xisca Sureda, María Sandín Vázquez

**Affiliations:** 1 Preventive Medicine Department, Hospital Universitario de Móstoles, C. Dr. Luis Montes, S/N, 28935 Madrid, Spain; 2 Public Health and Epidemiology Research Group, School of Medicine, University of Alcalá, 28801 Madrid, Spain; 3 Department of Epidemiology and Biostatistics, Graduate School of Public Health and Health Policy, City University of New York, 205 E 42nd St., New York, NY 10017, USA; 4 Tobacco Control Research Group, Institut d’Investigació Biomèdica de Bellvitge-IDIBELL, l’Hospitalet de Llobregat Avinguda de la Granvia de l’Hospitalet, 199 L’Hospitalet de Llobregat, 08908 Barcelona, Spain; 5 Centro de Investigación Biomédica en Red de Enfermedades Respiratorias (CIBERES), 28029 Madrid, Spain; 6 Department of Community Health and Social Sciences, Graduate School of Public Health and Health Policy, City University of New York, New York, NY 10027, USA

**Keywords:** gender, alcohol, gender violence, adolescents, risks, qualitative

## Abstract

The epidemiological information available in Spain and the Community of Madrid highlights two relevant facts regarding alcohol consumption: an increase in binge drinking in teenagers and a reduction/reversal of the gender gap, particularly at young ages. This article aims to describe some of the factors related to alcohol use in teenagers, especially those related to gender and risk perception. A qualitative study was designed with semi-structured interviews and a discussion group with students from the city of Madrid aged 14 to 18 years. A descriptive analysis of the content of the replies of 28 teenagers was conducted. The results show that alcohol consumption has an identity component, both in terms of transition to adulthood and gender role performance. Consumption is also associated with risks, especially those determined by gender inequality, which teenagers learn to manage as a means of survival in nightlife. Preventive campaigns typically lack a gender perspective and a focus on risk prevention. To reduce the prevalence of consumption and associated risks, these strategies need to be reformulated with a gender perspective.

## 1. Introduction

Alcohol is the most consumed psychoactive substance in the world. Its consumption was related to 5.3% of global deaths and 5.1% of the global burden of morbidity in 2016 [1]. This evidence makes alcohol one of the major health risk factors in the population aged 15 to 49 years [2]. In addition to its health impact, alcohol causes high economic and social costs [3].

The 2021 report on the use of alcohol, tobacco and illegal drugs in Spain [4] found that alcohol remains the psychoactive substance most commonly used by students aged 14 to 18 years, with the lifetime prevalence of alcohol consumption reaching 73.9%. Forty percent of students admitted having been drunk in the last year, and 23.2% did so in the last month. A comparative analysis with previous surveys [5] shows that the prevalence of alcohol consumption in the last month has maintained a decreasing trend since 2014. Data recorded in the Community of Madrid are similar [6]. Despite the decrease observed in recent years, these values continue to cause alarm, since teenagers are a particularly vulnerable group to the effects of alcohol and it is at these ages that poor health habits are usually initiated [7]. Alcohol consumption in adolescents is associated with significant negative biological and social effects, such as neurological affectation in the short and medium term with poor academic performance [8]; increased risk of sexually transmitted infections and unwanted pregnancies, and increased risk of physical and sexual aggression and other violent events [9,10,11]; increased consumption of other psychostimulant substances [12]; increased risk of chronic and long-term diseases such as cancer, cardiovascular diseases, etc. [13].

Additionally, two epidemiologically relevant phenomena have been observed in Spain in recent decades: an increase in binge drinking and a reduction/reversal of the gender gap in consumption. Binge drinking is defined as alcohol consumption that raises the blood alcohol concentration to 0.08 g/dL. Adolescents reach these levels by consuming 3–5 drinks in a 2-h period, depending on age and sex [14]. In recent decades, there has been a relative increase in women’s drinking, especially at younger ages, so that the historical gender drinking gap is narrowing and, at some ages, even reversing [4,6,15,16].

Both phenomena are related; the latest epidemiological reports at the state and regional level show that the prevalence of female alcohol consumption is higher than that of male consumption during adolescence, being especially striking in regard to heavy drinking in the younger age groups, at 14–15 years [4,6]. In our context, it is also relevant to understand the phenomenon of “botellón”, which consists of drinking alcohol in large groups (mostly young people) in open areas, squares or parks [17].

To understand these two phenomena, it is necessary to understand the determinants of alcohol consumption. These determinants include socioeconomic status [18,19,20,21,22], socio-cultural context [21,22,23,24], urban context [25,26,27], exposure to alcohol advertising in mainstream media [28] and social media [29,30,31,32] and accessibility [33,34,35,36]. Gender has also been identified as a determinant of alcohol consumption [23,37,38,39,40,41,42].

At this point, it is worth distinguishing sex—biologically determined—and gender—socially constructed [43]—as relevant variables in the study of the epidemiology of alcohol consumption.

The different effects of alcohol consumption according to sex have been studied, concluding that metabolic differences mean that women suffer greater effects of alcohol for the same amounts of consumption and a higher risk of dependence [42,44]. However, fewer studies have analysed the implication that gender may have regarding alcohol consumption and its potential negative effects. This is relevant because gender inequality conditions what is socially acceptable for women and men regarding drinking, with greater stigma for female users of psychoactive substances (legal and illegal) [23]. In addition, some negative consequences of substance use, such as the possibility of sexual assault, are clearly gender-related [11]. Chemical submission and, in particular, drug-facilitated sexual assaults stand out [45].

Gender inequality has also generated inequalities in diagnosis and access to preventive or therapeutic programmes because they have generally been designed from an androcentric viewpoint (focused on illegal substances or typically male consumption patterns) [38,46,47].

For this reason, the gender perspective should be present in any research or public health intervention. In the case of psychoactive substance use, it implies considering, in research, the influence of gender socialisation and inequalities in the forms of consumption and its consequences [48], and also taking this information into account when designing preventive policies or campaigns [41,49,50].

Studying alcohol consumption in the adolescent population from a gender perspective is particularly important. At this age, consumption begins and unhealthy habits can be established that continue into adulthood; on the other hand, it is a vital time for experimentation and learning to assume and manage risks, and, simultaneously, it is a stage in which gender roles are being shaped, generally marked by stereotypes inherited from previous generations [51]. In fact, some studies suggest that changes in the consumption patterns of boys and girls are related to the transformation of classical gender roles in recent decades [16,23,37,38,39,40].

This article aims to analyse, from a gender perspective, those elements related to alcohol consumption and the perception and management of the risks associated with it by adolescents in the city of Madrid, as well as to identify effective and contextualised proposals to prevent its consumption in terms of equity.

## 2. Materials and Methods

### 2.1. Research Design

The results presented in this article are part of a qualitative set of data obtained in a cross-sectional study using mixed methods to explore determinants of alcohol and tobacco use in secondary school students aged 14 to 18 years in the city of Madrid. The descriptive qualitative design [52] takes a phenomenological theoretical approach, and it is a recognised approach for investigating experiences in health research [53,54] with a gender perspective [55].The COREQ checklist was followed to improve the quality of the final report [56].

### 2.2. Participants

A theoretical, intentional, non-probabilistic sampling procedure was performed [57], seeking representativeness regarding variables that could influence participants’ responses: sex, age and socioeconomic status (SES), categorised by districts according to the deprivation index proposed by Gullón et al. [58].

The inclusion criteria were defined as being between 14 and 18 years of age, studying at an educational centre in the city of Madrid (public, subsidised or private) and having consumed alcohol at some point in their life.

The recruitment of respondents was carried out in two phases: in the first one, a flyer was disseminated in schools—at the door of the school in paper format and by teachers in digital format. This flyer contained a brief description of the study and a QR code leading to a form to fill in information about their consumption patterns and personal details to facilitate contact for the interview and discussion group phase.

In the second phase, the snowballing technique [59] was used to obtain key informants, with training in gender issues or participation in the school’s gender equality committee. Table 1 provides descriptive information on the sample of participants and the characteristics of the interviews.

The epidemiological situation related to the COVID-19 pandemic caused some of the interviews to be conducted via video call or phone call. The potential implications of the non-face-to-face interview format were considered by the research team [60].

### 2.3. Data Collection

General-phase fieldwork was conducted between April and September 2021, and in September 2022 with key informants. The main information-gathering technique was the semi-structured interview [61], and a discussion group was also conducted. Both were performed following a script (Table 2), agreed by the research team, which addressed the main thematic blocks defined according to the study objectives. Both the discussion group and the interviews were recorded, after obtaining verbal informed consent from the respondents, and transcribed literally by personnel not involved in the research. The data collection process ended when the different categories of analysis were considered to be adequately saturated [62].

Twenty semi-structured interviews and a non-mixed discussion group with 5 participants were conducted to address the general theme of teenage alcohol consumption. The discussion group was non-mixed in order to facilitate the girls’ discourse and to avoid confrontation due to the sensitivity of the topic. It included girls of a single SES because, until then, no differences had been observed in the discourse with respect to SES and in order to facilitate participation due to proximity, as it took place in their own neighbourhood. In response to the appearance of emerging themes related to gender and alcohol consumption, the study was expanded with 3 interviews with teenagers who, either because they participated in their school’s gender equality committee or because of their gender training, were considered key informants.

### 2.4. Data Analysis

A thematic descriptive analysis with a phenomenological approach [52,53] was performed using the ATLAS.Ti software (Scientific Software Development GmbH. Qualitative Data Analysis. Version 8.0. Berlin, Germany, 2018). The analysis was structured based on the code tree previously developed by the research team and modified in response to the appearance of emerging categories or relevant concepts during the review of the information obtained in the field work. The process carried out by the researchers was as follows: coding the data (trying to identify objects of concern, relationships, concepts, processes, etc.), looking for themes related to the objectives, connecting the themes with a pattern of meaning, revisiting previous texts to revise the initial coding and allowing new categories to emerge inductively from the text. In order to increase the quality and validity of the study and avoid possible bias in the coding, it was performed independently by three researchers using a triangulation process [57,63]. This process also improves the dependability. We also decided to use methodological triangulation [64], choosing semi-structured interviews and a discussion group as the main techniques because both techniques shed light on the relationship between personal and social discourses, and because differing data sources have produced similar findings on the same issue and thus a consistent narrative about the issue under examination. In order to increase the confirmabiliy, we followed the COREQ checklist [56].

## 3. Results

Twenty-three individual interviews were conducted with teenagers. A total of 13 girls and 10 boys were interviewed, 11 aged 14–16 and 12 aged 17–18. Of these, 8 studied in low-SES neighbourhoods, 10 in middle-SES neighbourhoods and 5 in high-SES neighbourhoods. The discussion group consisted of five girls, three 18-year-olds and two 17-year-olds, students in the same middle-SES neighbourhood (see Table 1). Interviews ranged in duration from 30 to 79 min. The discussion group lasted 125 min.

Despite being included as a stratification variable, the interviews conducted revealed no differences by SES in the gender approach or risk prevention, so no differential analysis of the results by this variable was performed.

### 3.1. Peer Consumption, Identity and Influence

Our respondents discussed their first alcohol use and reasons for experimenting with alcohol. Curiosity appears often, but highlights a certain sense of inevitability, as something to be expressed at this life stage as a transition to adult life.

*After all, we all wanted to say: “Bah, this is what they do, I’ve seen older people do it” And wanting to feel older than you are, and that curiosity…*

(A01, boy, 18 years old, middle SES)

*My female friends in my home town […] are all older than me, all of them. I’m the youngest and I’m the only one of my age, so when I was 14, because they were already drinking alcohol, obviously Larios, what we said, so I said, okay, well I’ll start. But, well, I remember that summer I was drinking maybe a super-low-alcohol rum and coke, but in a very scared way.*

(GD05, girl, 18 years old, middle SES)

“Initiating friends” appear in the accounts regarding the participants’ first alcohol use, who are close persons who have already experimented with alcohol and introduce the inexperienced to consumption. In this regard, it should be noted that, at these ages, it is common for teen girls to have friendships with persons older than them, favouring an earlier start in consumption.

*Right at that point in my life I was with people around me who were older and who were already starting to do “botellón, botellón, botellón”, over and over, and I didn’t drink and […] there came a time when they said “but come on, today you will drink”. And I was like, “Well, if I have no other choice” [laughs].*

(GD02, girl, 18 years old, middle SES)

With regard to differential consumption by gender, there is a fairly widespread perception that alcohol has a greater effect in girls, but there is no unanimity as to whether this occurs because they consume a larger amount than boys or whether it is due to physiological differences (greater effect with the same consumption).

*Well, I think women we kind of retain alcohol more or something like that. I don’t remember exactly.*

(A19, girl, 16, low SES)

*Fuck!, I really […] prefer that she doesn’t… that the girls don’t drink, because I know alcohol can have a much worse impact on them and they can end up doing things they don’t want to. And on top of that… I don’t know, they’re much more vulnerable*.

(A15, boy, 17, low SES)

Several male respondents considered that some girls engage in more uncontrolled consumption or tend to exaggerate the effects of drunkenness (according to them, they drink “worse”), as opposed to the perception of some female respondents that it is boys who tend to lose control.

*I have noticed that boys take longer to get drunk and they also lose it a lot more. In other words, in my group the girls tend to be calmer, and so, and the boys already start with more partying, more “come on, and that, I get drunk, to see how it is, I don’t know what”, and they do competitions, well, come on, boys, relax!*

(A11, girl, 18 years old, low SES)

*I’ve seen 14 and 15-year-old girls who were so pathetic. It was ridiculous to see that, to see them lying there on the ground, laughing there and it wasn’t funny at all, “you’re pathetic.” […] The thing is that girls usually drink until they are out of it or feel really sick, and boys don’t. Some do, too, but they don’t usually.*

(A07, boy, 16 years old, middle SES)

*And there are also a lot of girls who come to parties just to meet people and sort of pretend to get drunk, and that’s not good, either […] We know when a person is drunk and when they are not. And a person comes here to put on a show, and it’s tiresome, it ends up being annoying, you know? You end up saying to her, “Slow down a little, relax a little, you’re really just playing the fool here.”*

(A15, boy, 17, low SES)

This comment shows how the social interpretation made about the consumption of boys and girls is also unequal. The expectation of compliance with gender stereotypes marks what is or is not reprehensible for girls or boys. One of the respondents questioned the different social assessment of consumption according to gender.

*Boys have a lot more freedom from a young age. It’s a reality, it should change, but it’s a reality. And girls always wear that label saying “well, ladies should not drink, ladies should do this”. And, well, it’s a backward mindset, but, well, we’re fighting against that little by little.*

(A13, girl, 17 years old, middle SES)

*Maybe a little bit of the tradition […] that a man has always had the opportunity to drink and it wasn’t frowned on, and yet a woman who drinks and maybe enjoys alcohol has been kind of a more repressed thing throughout history, I understand*.

(A22, girl, 15 years old, middle SES)

These comments could illustrate some of the assumptions about reducing/reversing the gender gap in consumption; however, another respondent believed that the increase in consumption by girls is due to other reasons: a means of being accepted by boys.

*Now it’s like that, you’re looking a lot for male approval which is something that makes me feel sick. Because on top of that they look for it in very despicable ways […] So it’s “if the guys drink more I’m going to drink a lot too to be accepted.” And I don’t know, just that you have to drink a lot to be accepted…*

(A21, girl, 15, middle SES)

This profile of girls is referred to as “*pick me girls*”, who wish to make it clear that they are “not like the other girls” and modify their behaviours, seeking male approval. She believes that female empowerment should not necessarily increase consumption but involves acting on one’s desires.

*You see, me and my group of female friends who are very open-minded and very cool, more than wanting to break roles is… “I am like that and I don’t need to show it to anyone”. And then these “pick me” girls […] is that I’m pretty sure it’s “I want to be funny because that’s how I’m going to get in better” [in the boys’ group] and drink and drink so he says “oh, look how she drinks”*

(A21, girl, 15, middle SES)

This respondent believes that boys also adopt certain attitudes (sexist comments, taking certain risks, etc.) to adapt to the pattern of masculinity. That is, they act in a certain way to meet gender expectations.

*A friend and I talk about “the pack” […] and it’s the social status boys have among boys. And for some reason that we haven’t been able to figure out yet, among themselves they have to be more annoying, more macho, because that’s it, sexist and even homophobic, which then they’re not at all, you know, but among other boys they have to be more macho. But… it’s really awful!*

(A21, girl, 15 years old, middle SES)

### 3.2. Consumption, Risk Perception and Survival

When starting consumption, it is important to learn how to manage risks. Although most teenagers do not perceive the risks in the medium or long term, according to their accounts, it seems clear when facing consumption in large quantities there is a dangerous consumption threshold that should not be reached. Control strategies are developed for this, which are learned initially from friends who are more used to drinking and later from their own experience, so that each one learns how and how much they can drink.

*I am very afraid, for example, to try a new alcohol that I have never tried and to drink a lot of that alcohol. In other words, what I would say is to try […] a little to see how they feel […] and, well, increase the dose they take a little, so little by little, to see how they are feeling. Like with joints, it seems the same.*

(A08, girl, 16 years old, high SES)

*I interpret it as maybe for something very serious to happen to my liver, I have to drink almost every day and large amounts, or even small amounts, but every day. And I don’t drink that, you know? I drink maybe very occasionally, or whenever… So, I know I have to be in control, but for now I don’t worry*.

(A19, girl, 16, low SES)

When control is not enough, peer care appears; the friendship group takes care of the “uncontrolled” person to prevent the situation from escalating and new risks or complications arising. This is a widespread practice and, although sometimes rotating, there tend to be profiles of carer friends, especially non-drinkers. In exploring the risks linked to gender, the concept of risk appears in testimonies from a relationship point of view, i.e., the effect that consumption may have on the boys (known and unknown) who are present with them, or even on their ability to protect themselves from harassment or abuse. In addition, they are aware that consent to sexual intercourse is socially considered unnecessary if they have drunk alcohol, which becomes problematic because it justifies non-consensual intercourse in the context of alcohol consumption.

*You see, he was a “friend” in quotation marks, that kind of friend, right? So, well… once we were alone and, well, he did that, and he was like “hey, calm down,” and I had to talk to him like 2 or 3 times so that… that he lost it. Then we talked about it and he asked me for forgiveness and so on, and that was, I think because he was also feeling a little bad, I don’t know. [I felt] uncomfortable. But well, then we talked about it, and like… I mean, like we’ve done things several times before, because it was like, well… “Well, okay, for one time”*.

(A18, girl, 15, low SES)

*A girl from the neighbourhood, there was a guy who was little drunk and he forced her to suck his penis, but she got hysterical and screamed and they heard her. […] And we have all heard news of “well she was drunk and he insisted, insisted and insisted and in the end, well, ok.” But that’s not a yes!*

(A21, girl, 15 years old, middle SES)

In fear of possible abuse by them, they use various strategies, one of the most mentioned being control of their own consumption.

*I’m afraid of alcohol when it’s an issue… when it becomes an issue that’s a bit sexual, because sometimes you don’t know […] if someone wants to and you don’t, it’s difficult to communicate and all that. And the boys, in general, at least in my experience and in many other girls’, I think sometimes they lose it a lot also and they don’t listen to you when you talk to them. […] So, on the subject of alcohol, that’s why I don’t usually drink too much, because I feel like they’re going to do something to me.*

(A18, girl, 15, low SES)

*In the women around me what I see is more responsible consumption […] I understand that it’s already a question of safety, of saying “and what can happen to me now if I suddenly drink and lose control of the situation and someone comes to do something to me?” it changes completely, when you talk about gender it’s a different matter […] They don’t have to worry about whether a guy is going to come and come from behind and do anything or they’re going to shout at them in the street or anything can happen to them.*

(A22, girl, 15 years old, middle SES)

Control does not protect them from the attitudes of others but makes them feel less vulnerable and better able to react in these situations. This individual strategy is complemented by a collective one: care for each other.

*Luckily, in the circles I move in there’s a lot of sisterhood. So if I see a female friend who is suddenly very down and has drunk too much, then there I go and 7 more go and we are there with her*

(A22, girl, 15 years old, middle SES)

*We’ve been in some places and we’ve seen maybe a guy who was getting very annoying and so on, but he never got to do anything because, well, we’ve been able to stop him. […] It happened to me once, a very annoying boy, I cut him short from the start […] that was it, but it happened to a friend, one started behaving a bit bad and we had to stop him, there were 3 of us.*

(A09, girl, 16 years old, middle SES)

Some of the girls stated that they feel safer when the group is large and especially if there is a male presence. Although they feel more secure, they continue to feel that they are objects of desire/consumption. In addition, they interpret that, in these cases, the harassment that they receive is reduced because the boys are respected, rather than the girls themselves.

*If we go to a place and we’re going to drink, […] not always, but we try at least, well, to have a boy, because I don’t know, in the end they respect you a little more. […] maybe there are 4 or 5 of us girls and they are yelling at us, they are saying things to us, and maybe you go with a boy and nothing happens.*

(A09, girl, 16 years old, middle SES)

*It’s very sad, but the fact that a man goes with us helps a lot, because it’s true that when we were Interrailling we met with […] male friends of my female friend. We went out with them in those places and it was where we got the least harassment practically, where we barely got harassment, you know. And the three of us agreed that it was because we were accompanied by men […] They don’t respect you, but they respect the man, and because they respect the other man, they don’t do anything to you. […] It’s disgusting, really. But of course, by the next time we go on a trip, maybe we’ll… I mean, it’s safer. It’s crap, but well.*

(A23, girl, 18 years old, middle SES)

Another issue of concern in some cases is the fear of drug-facilitated sexual assault by introducing substances into their drinks to reduce their reaction ability.

*I have heard many times especially men saying, “I have decided to get this girl drunk to hook up with her” and they see it as normal, as if it were not a problem.*

(A23, girl, 18 years old, middle SES)

*When I go to “botellón” she says (my mother) “please if you drink […] drink from your glass, don’t let someone see it, fill it up yourself, see if they are filling it up…” […] So they don’t put anything in it.*

(A21, girl, 15 years old, middle SES)

*I don’t like people and even less when I don’t know them and it’s usually: I go, I see the scene and I leave. But female friends of mine who do stay [where they’re on “botellón”] are more aware of covering the glass just in case, later we don’t think anything will happen, but it’s that “just in case.”*

(A21, girl, 15 years old, middle SES)

Caring for each other again appears as a protective measure when alertness has not been sufficient:

*The idea that “as soon as you feel dizzy, you look for us”. Even friends of mine say “you feel dizzy, you look for me”.*

(A21, girl, 15 years old, middle SES)

*My sister was at a festival this summer and went with her friend and his friend’s friends and she thinks they put something in her drink […] She remembers her friend telling her “I’ll take you to the tent because you’re feeling so bad”*

(A21, girl, 15 years old, middle SES)

One of the key moments in nightlife is the return home, when being alone and drunkenness (our own, but especially others’) can increase the risk of assault. Again, control and care strategies are combined to allow individuals to reach home safely.

*You see, there are times that I have maybe controlled myself, because I have said that I don’t want to go feeling bad, because maybe I have to go home alone or something and I think boys have never had to say “well I won’t drink because I have to go home alone”.*

(A09, girl, 16 years old, middle SES)

*When it’s time to go home I try to be as sober as possible so that I can cope better with what might happen. So that I can act in a more conscious way.*

(A23, girl, 18 years old, middle SES)

*And it’s no longer what we consume, but what other people consume, so late at night going with the keys in hand and to be alert to “see who I come across”.*

(A22, girl, 15 years old, middle SES)

*You put a key between your fingers, in case, I don’t know, anything can happen. And to have it on hand as well so you can get in as quickly as possible.*

(A23, girl, 18 years old, middle SES)

*And when it comes to returning home “you call me, I don’t know what, talk to me on WhatsApp” […] Being in continuous contact, friends of mine who leave, call me and are all the way talking to me […] Luckily, it’s also a gender consciousness that if you know how to do it, it becomes a habit. Friends of mine who already know you have to be alert when a woman returns home or just to say “hey, call me or whatever”.*

(A22, girl, 15 years old, middle SES)

Despite the different precautions that they take, assaults continue to occur. According to our respondents, the social assessment of alcohol consumption in these situations is different depending on gender. In the case of boys, consumption tends to be seen as a mitigating factor, whereas, in girls, it is often a blame-placing factor.

*You see, this girl is loved by a lot of people, so they reacted more, but for this girl, you know, maybe in another moment I’m sure there’d be more than one comment of “what was she wearing?”, even in some way of seeing her as blameworthy, like “she was probably drunk”, but what difference does it make!!*

(A21, girl, 15 years old, middle SES)

*If a man had been drunk and committed sexual abuse, he’s going to be less guilty… he’s going to be… he’s going to be justified because he was drunk… Instead, women are being criminalised for drinking*.

(A23, girl, 18 years old, middle SES)

### 3.3. Proposals to Prevent Alcohol Consumption and Associated Risks

The respondents expressed proposals on how to prevent the risks associated with alcohol consumption, both biological and relational. They see a change in social discourse, which speaks in a more accepted way of the need for respect for women, although they feel that it needs to be promoted further.

*Well, with our male friends we do talk about it and on top of that they feel very uncomfortable because it’s like “no, I respect you, I respect you!” and we say “but you keep in mind that only yes is yes, and no is no” “so, please leave me alone” [Laughter]*

(A21, girl, 15 years old, middle SES)

*I think now people are changing it, it’s the talk at home that it’s not “don’t wear this skirt”, that it’s “respect the girls” […] I think when my brother goes out [my parents] will say “respect them, and so on…”*

(A21, girl, 15 years old, middle SES)

For the girls, the key is education, which must be present in all areas: family, group of friends, schools, audiovisual media, etc.

*With education, with a lot of education, and from within and from now on. That is, from public institutions, from secondary schools, primary schools…*

(A22, girl, 15 years old, middle SES)

*Education in secondary schools basically, because at home not all parents are going to educate their children like that, so it would have to be established in a way so that all children could have access to that education about gender.*

(A23, girl, 18 years old, middle SES)

With regard to talks on consumption in educational institutions, some consider that they should start at a younger age, so that they occur before consumption begins, as well as that they should use formats more adapted to their methods of communicating and include content that meets their needs, such as risk reduction or gender-specific training so that assaults are no longer a constant threat (in leisure and consumption settings and outside them).

*On the last day of our university entrance exams, some boys came up and told us: “Hey, would you like us to give you a talk on how to drink responsibly?” […] Until that moment I had no idea. And, of course, maybe it’s very difficult for you to tell me, “Hey, don’t drink or don’t smoke” and convince me of that. But if I’m already more aware of the consequences that it can have and […] if I do it, how to do it carefully, then I’ll think about it more, you know?*

(GD03, girl, 18 years old, middle SES)

*Tell me what to do if I drink alcohol, how to drink it responsibly, and how to figure out how to tackle problems if those problems ever come in my life. Because they will come and I’m not going to be able to react. […] Why are you telling me […] that it is very bad to drink? Better tell me “well look, you’re drinking, let’s get straight to the point.”*

(GD02, girl, 18 years old, middle SES)

*Inform us a little at a statistical level what is happening and maybe the talks give rise to debate so maybe the voice of each student comes out, they express their opinion, ideas, experiences they have and then you summarise it. […] in these situations, what can we do? Talk a little bit about that individual responsibility as well.*

(A22, girl, 15 years old, middle SES)

*Not having received a feminist education, men are going to act in a way…that they don’t care how they make other women feel.*

(A23, girl, 18 years old, middle SES)

*The idea would be, hmm, to incorporate a little bit of this, this gender perspective, right? Because at the end of the day in all the situations of our life that we find ourselves in, this is going to be a little bit determined, and of course, when we talk about alcohol and its consumption, well, to introduce a little bit of these things.*

(A22, girl, 15 years old, middle SES)

## 4. Discussion

The results of this study allow us to understand the complexity of alcohol consumption in teenagers. In addition to being associated with leisure, it has a strong identity/peer influence component. On one hand, the start of consumption and its normalisation is considered an inevitable fact that marks the passage to adulthood; on the other, the existence of positive expectations regarding consumption tends to increase it [65]. Therefore, consumption not only implies growing up; it is also considered relevant for belonging to a peer group and for having fun, so that consumption at these ages dominates leisure and socialisation spaces. Indeed, group pressure and social contagion appear in many of the accounts collected and in other research studies [22,37,40,66].

Consumption patterns are also related to gender identities. In recent years, surveys in Spain have indicated that alcohol consumption is becoming equal between men and women at a young age and that binge drinking is increasing, especially among young women. Our results indicate that this reduction/reversal of the gender gap may be related to the transformation of roles traditionally associated with men and women, as indicated by some studies [37,39,40,67], which also identify that consumption occurs in a public space (“el botellón”) as an additional transgressive element. In contrast, one of our respondents suggested that the increase in consumption in girls may be due to the search for acceptance by boys, mentioning the phenomenon of “pick me girls”, which has been identified as a form of internalised sexism by promoting competition among women [68].

However, this shift in consumption patterns does not yet appear to correspond to a shift in social valuation. Socially, female consumption continues to be disapproved of, as we have seen in the comments of our respondents that agree with the results of other studies [40,66]. This social disapproval occurs especially if consumption is disproportionate or if it includes sexual behaviour usually disapproved of in women, such as a desire to engage in casual sex.

An important point to understand in terms of teens’ drinking motivations and their imagination regarding alcohol and gender roles is marketing. In recent years, the alcohol industry has altered the ways in which it addresses women by adapting to the current socio-political context with feminist and equality messages [69,70,71]. This evidence is in line with the testimonies of our respondents, who associate alcohol consumption with empowerment and the conquest of traditionally masculine spaces.

In a recent study in 2021, the authors analysed how alcohol marketing presents alcohol use as a combination of traditional, post-feminist and feminist femininities with more intersectional representations, in order to attract the largest number of women, especially younger women. All this accounts for the commodification of the message of female empowerment, as alcohol consumption increases structural inequality and impacts women’s health, with women being the most vulnerable to the consequences of their consumption [70]. In this regard, public policies to regulate advertising are a key element in preventing consumption, especially in the teenage population [33].

Another factor determining consumption, particularly from a gender perspective, is risk taking and risk management—both those derived from one’s own consumption, and those connected with consumption by others and interpersonal relationships. Although the general perception of risk is relatively low and focuses more on immediate biological consequences (linked to binge drinking) or very long-term consequences (linked to daily or very regular consumption), drinking is associated with risks, and, therefore, at this vital stage, it is necessary to learn how to manage them and minimise their impact.

The idea of controlling alcohol consumption is central to the discourse, the aim being to achieve the pleasant effects of alcohol without exceeding this hypothetical risk threshold or the amount at which one is considered to have control of oneself. However, these amounts are highly dependent on individual susceptibility and often are related to binge drinking, with no perception that this could have any impact on a biological level [24,72,73]. This idea of control has been promoted by the alcohol industry, promoting the notion of “moderate or responsible consumption” as risk-free, when it is known that there is no level of alcohol consumption that is safe for the health of those who consume it [74]. Only a zero level of consumption minimises health risks, so the perceptions of teenagers must be taken into account in order to formulate suitable preventive programmes.

Exposure to consumption risks is unequal according to gender. Girls are at a greater risk than boys of suffering from a number of unpleasant and dangerous situations resulting from their own consumption and consumption by others [9,12,37,73,75]. These latent risks (many have not experienced them but are aware that they could happen at any time) generate protective attitudes on their part, both individually and collectively, so that they can enjoy leisure and socialisation safely.

As individual strategies to reduce risks, our informants mentioned reducing their consumption in order to be able to react to possible aggression, watching their drinks to avoid being drugged or using their keys as a defence against aggression. Peer care, which is also generally practised when consumption is excessive, takes on a special dimension in the face of gender violence in leisure settings. Female friends appear when harassment occurs; they accompany or monitor each other on their journeys home and are alert to potentially more vulnerable situations, such as drug-facilitated sexual assault: “as soon as you feel dizzy, you look for us”. Implicit in this commentary and the rest of the accounts is the idea of being at risk as objects of desire and consumption.

A study carried out in the context of the “botellón” analyses how boys perform their gender roles (masculinity) through two strategies, predation (with proactive attitudes towards violence) and conservation (with defensive techniques based on care and protection). It also mentions that, in nightlife, girls are a consumer product for boys [76]. This is apparent when respondents claim to be less likely to be assaulted when accompanied by boys, because the boys are respected. In this scenario, teenage girls are forced to opt for preservation if they wish to enjoy leisure in safe conditions.

Care and self-care in these situations are so normalised that individual responsibility to avoid assault is placed on teenage girls. This can be seen in the perception that many teenage girls have about how women are judged when they are assaulted in leisure settings and have consumed a substance, and also in the reality that, in most cases, they receive advice at home on how to protect themselves, while the recommendation to boys to respect girls and not assault them does not seem to be widespread [75].

Therefore, just as levels of consumption are becoming equal, freedom in the occupation of leisure spaces does not occur on equal terms, so that the balance in consumption combined with the imbalance in risks in interpersonal relationships may be translating into the greater exposure and vulnerability of women to situations of harassment and abuse, from which they are not exempt even when modifying their behaviours [12,37,73,75].

In addition to modifying their behaviour as a method of protection, our informants advocate for education in their environment by raising awareness among their friends, families, etc. However, they consider that this is not enough and suggest that these subjects should be dealt with in a structural way, both in families and in educational institutions. Prevention and promotion strategies for healthy leisure should therefore focus on risk reduction in a global manner and from a gender perspective, understanding that many risks from consumption are related to gender inequalities, such as sexual violence in leisure and substance use settings [75,77].

Following the suggestion of one of our informants, it would be important to identify and work on individual attitudes to promote individual responsibility for collective change—understanding that it is a social problem, but that we are all responsible for cultural change. Girls recognise themselves as potential victims and are already modifying their practices; however, potential aggressors need to recognise themselves as such in order to work on their attitudes. This would also help to eliminate the sanctioning judgement that falls on women, as the only ones responsible for protecting themselves from “ghost” aggressors [75].

Finally, campaigns to prevent consumption and reduce risks should start at a younger age, anticipating the initial consumption among teenagers, in order to prevent/delay it [77]. They should also adapt their formats and content to the communication methods of this group and their perceived needs, including clear and sufficient information on the risks associated with consumption. Gender education, as already mentioned, should be a key aspect in any prevention and health promotion campaign.

### Limitations and Recommendations

Among the possible limitations of the study is the need to resort almost exclusively to the interview technique, sometimes performed by telephone or Zoom, to the detriment of discussion groups and face-to-face interviews. Although the aim was to generate a climate of trust, and care was taken to ensure that interviews were conducted at a time when students had privacy to speak without being censored, the lack of physical presence could have influenced the discussions (since the specific context of the interviewee could not be known). On one occasion, it seemed that the adolescent was being listened to by a relative and did not respond naturally, but, in the rest of the interviews conducted remotely, the interviewers felt that the necessary rapport had been achieved to ensure the quality of the information.

A broader study would allow us to further explore the discussions about sexual assault from the perspective of boys, helping to better target preventive campaigns. It would also be helpful to identify changes in consumption patterns, associated risks and protection strategies across the different age groups that comprise adolescence.

## 5. Conclusions

Alcohol consumption in teenagers is a complex issue with multiple social meanings. Appropriate theoretical and methodological approaches are needed to understand the changes taking place in the prevalence of consumption, by type of consumption (binge drinking) and by gender differences (reduction/reversal of the gender gap).

Such important phenomena as identity building, peer influence or risk management (especially fear of sexual assault) determine how, how much and why alcohol is consumed. In addition to recognising the most relevant identity elements of adolescence (the group, the transition to adulthood, etc.), it is essential to incorporate the gender perspective into the analysis, since gender socialisation determines consumption by identity, both through breaking stereotypes and imitation, and by risk management.

Gender inequality and sexual assault are not only an equality issue but also a public health issue; therefore, the gender perspective should be included in any health prevention and promotion programme, especially those addressing recreational substance use. In addition, policies would be needed to regulate alcohol advertising—in particular, advertising aimed at women and adolescents that associates femininity and success with alcohol consumption, misrepresenting the associated risk.

## Figures and Tables

**Table 1:** Summary table of informants and interviews.

Informant Identification	Age	Sex	Socioeconomic Level (SES)	Type of Interview
A01	18	M ^1^	Middle	Face-to-face interview
A02	15	M	High	Video call interview
A03	18	F	High	Phone call interview
A04	17	M	High	Face-to-face interview
A05	17	F	Middle	Phone call interview
A06	18	M	Middle	Video call interview
A07	16	M	Middle	Phone call interview
A08	16	F	High	Phone call interview
A09	16	F	Middle	Phone call interview
A10	14	F	Low	Phone call interview
A11	18	F	Low	Face-to-face interview
A12	17	M	Low	Video call interview
A13	17	F	Middle	Video call interview
A14	17	M	High	Phone call interview
A15	17	M	Low	Phone call interview
A16	16	F	Low	Face-to-face interview
A17	15	M	Low	Face-to-face interview
A18	15	F	Low	Face-to-face interview
A19	16	F	Low	Face-to-face interview
A20	16	M	High	Face-to-face interview
A21	15	F	Middle	Face-to-face interview
A22	15	F	Middle	Face-to-face interview
A23	18	F	Middle	Video call interview
GD01	17	F	Middle	Face-to-face discussion group
GD02	18	F	Middle	Face-to-face discussion group
GD03	18	F	Middle	Face-to-face discussion group
GD04	17	F	Middle	Face-to-face discussion group
GD05	18	F	Middle	Face-to-face discussion group

M ^1^ = Male F = Female.

**Table 2:** Interview topic guide for key informants.

Thematic Area	Questions
**First consumption** **Context** **Motivations**	Do you remember the first time you drank? Where? Who were you with? What did you do? How did you feel? Why did you try it?
**Consumption patterns** **Individual** **Differential consumption** **by gender**	Have you continued drinking? Why? When do you do it? With whom? Why do people around you drink? Are there differences in the consumption of boys and girls in your environment? How do they drink? Why do you think this happens? What is the effect of alcohol on boys and girls? Why do you think this is so?
**Social influence** **Group pressure** **Social judgement**	What are leisure plans with your friends like? Is drinking common? What if someone doesn’t drink? Do you think being around people who drink influences you? How is consumption by boys considered? And by girls? Do you think there are differences? Why?
**Risks** **Risk perception** **Risk management** **Unpleasant experiences (gender)**	How do you think drinking alcohol can affect your health? And your life in general? What consequences can consumption have on your friends? Is it different for your male friends than for your female friends? How do you avoid risks? How do you learn it? Do you receive information about this at home? What kind? Have you had any unpleasant experiences related to consumption (yours or others)? Can you tell me what happened? How did you feel? How did you solve it? Why do you think it happened? Has it happened to you on other occasions? And other people? Do you talk about these experiences among your female friends? Is it the same for boys and girls? How do you avoid these situations? Do you get advice at home? And your male friends and female friends?
**Prevention** **Preventive campaigns** **Proposals**	Do you know of any preventive campaigns about drinking? Where? What do they talk about? How do you value them? What do you think a campaign for young people not to drink should be? And to avoid the risks associated with consumption? What content should it include? How would it work best (format)?
**Conclusion**	Is there anything that you did not mention in the interview that you feel should be recorded?

## Data Availability

The data associated with the paper are not publicly available but are available from the corresponding author upon reasonable request.
